# The new insight into extracellular NAD^+^ degradation‐the contribution of CD38 and CD73 in calcific aortic valve disease

**DOI:** 10.1111/jcmm.15912

**Published:** 2021-06-18

**Authors:** Patrycja Jablonska, Barbara Kutryb‐Zajac, Paulina Mierzejewska, Agnieszka Jasztal, Barbara Bocian, Romuald Lango, Jan Rogowski, Stefan Chlopicki, Ryszard T. Smolenski, Ewa M. Slominska

**Affiliations:** ^1^ Department of Biochemistry Medical University of Gdansk Gdansk Poland; ^2^ Jagiellonian Center for Experimental Therapeutics Jagiellonian University Krakow Poland; ^3^ Department of Cardiac & Vascular Surgery Medical University of Gdansk Gdansk Poland; ^4^ Department of Cardiac Anaesthesiology Medical University of Gdansk Gdansk Poland

**Keywords:** aortic valve, calcific aortic valve disease, ecto‐enzymes, mononucleotide nicotinamide, NAD

## Abstract

Nicotinamide adenine dinucleotide (NAD^+^) is crucial for cell energy metabolism and many signalling processes. Recently, we proved the role of ecto‐enzymes in controlling adenine nucleotide–dependent pathways during calcific aortic valve disease (CAVD). This study aimed to investigate extracellular hydrolysis of NAD^+^ and mononucleotide nicotinamide (NMN) in aortic valves and aorta fragments of CAVD patients and on the inner aortic surface of ecto‐5′‐nucleotidase knockout mice (CD73−/−). Human non‐stenotic valves (n = 10) actively converted NAD^+^ and NMN via both CD73 and NAD^+^‐glycohydrolase (CD38) according to our analysis with RP‐HPLC and immunofluorescence. In stenotic valves (n = 50), due to reduced CD73 activity, NAD^+^ was degraded predominantly by CD38 and additionally by ALP and eNPP1. CAVD patients had significantly higher hydrolytic rates of NAD^+^ (0.81 ± 0.07 vs 0.56 ± 0.10) and NMN (1.12 ± 0.10 vs 0.71 ± 0.08 nmol/min/cm^2^) compared with controls. CD38 was also primarily engaged in human vascular NAD^+^ metabolism. Studies using specific ecto‐enzyme inhibitors and CD73−/− mice confirmed that CD73 is not the only enzyme involved in NAD^+^ and NMN hydrolysis and that CD38 had a significant contribution to these pathways. Modifications of extracellular NAD^+^ and NMN metabolism in aortic valve cells may be particularly important in valve pathology and could be a potential therapeutic target.

## 1 INTRODUCTION

**FIGURE 1:**
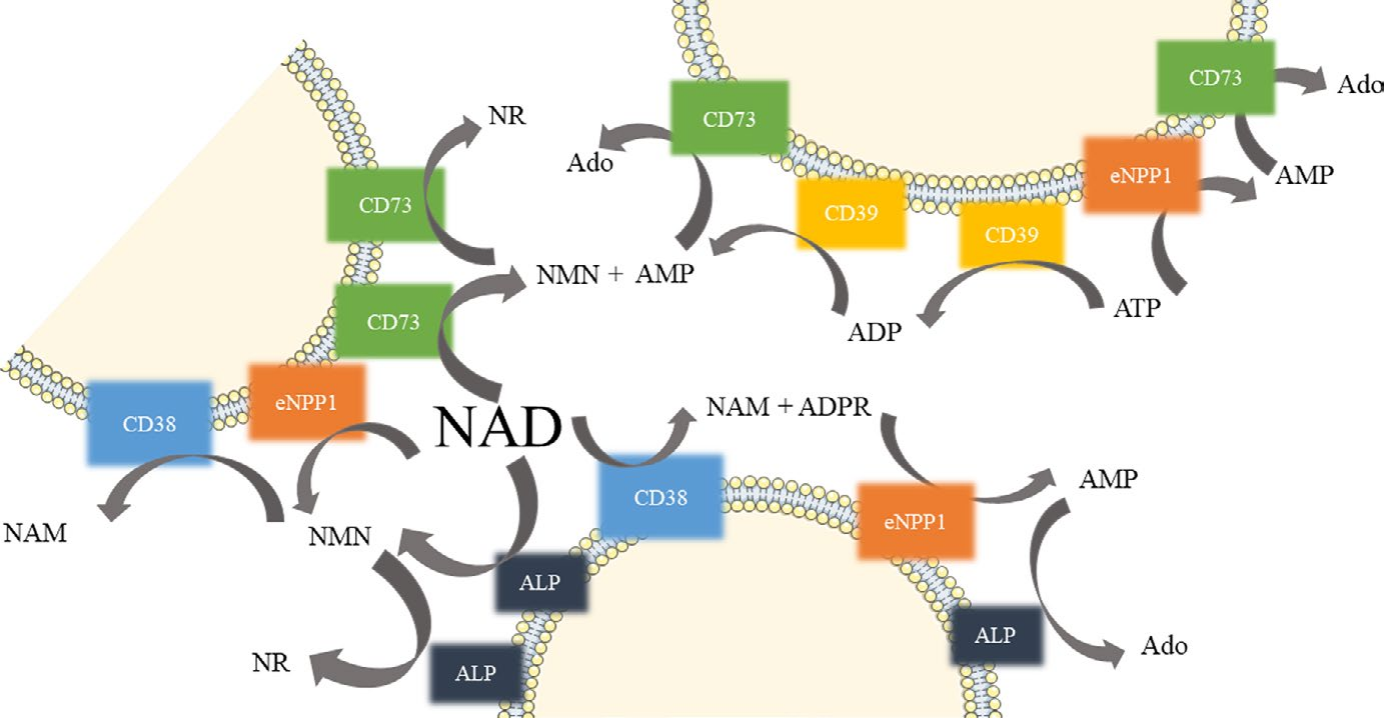
Schematic pathways of extracellular NAD^+^ and ATP hydrolysis and the interrelations of the ecto‐enzymes. Ado, adenosine; ADP, adenosine diphosphate; ADPR, ADP‐ribose; ALP, alkaline phosphatase; AMP, adenosine monophosphate; ATP, adenosine triphosphate; CD38, NAD^+^‐glycohydrolase; CD39, ecto‐nucleoside triphosphate diphosphohydrolase 1; CD73, ecto‐5′‐nucleotidase; eNPP1, ecto‐nucleotide pyrophosphatase/phosphodiesterase 1; NAD, nicotinamide adenine dinucleotide; NAM, nicotinamide; NMN, nicotinamide mononucleotide; NR, nicotinamide riboside

Nicotinamide adenine dinucleotide (NAD^+^) plays an important function as an electron carrier and redox cofactor for metabolism and energy production. It serves as a substrate for enzymes involved in healthspan and longevity and its degradation is a key element of a wide range of signalling pathways.^1^ NAD^+^ can be released from cells by lytic and non‐lytic mechanisms and metabolized on the cell surface. Extracellularly, NAD^+^ is catabolized to nicotinamide mononucleotide (NMN) and further to nicotinamide riboside (NR) by ecto‐5’‐nucleotidase (CD73; EC 3.1.3.5).^2^, ^3^ NAD^+^‐glycohydrolase (CD38, NADase; EC 3.2.2.5) is involved in the NAD^+^ catabolism to nicotinamide (Nam) and ADP‐ribose (ADPR).^4^ The degradation of NAD^+^ to NMN and adenosine monophosphate (AMP) can also be mediated by ecto‐nucleotide pyrophosphatase/phosphodiesterase 1 (eNPP1; EC 3.1.4.1).^5^ AMP is further hydrolysed to adenosine by CD73. Non‐specific alkaline phosphatase (ALP; EC 3.1.3.1) also takes part in nucleotide catabolism. As an enzyme with wide specificity, it breaks off the NAD^+^ phosphate group to form NMN followed by NR (Figure 1). Recently discovered, NADase sterile alpha and TIR motif containing 1 (SARM1; EC 3.2.2.6) is a new class of NADase that cleaves NAD^+^ into Nam, ADPR and cADPR via its TIR domain.^6^ In turn, the non‐hydrolytic reaction includes mono‐ADP‐ribosyltransferase (ART; EC 2.4.2.31) activity and leads to the formation of Nam and covalent protein modification.^7^

The same enzymes involved in extracellular nucleotide metabolism (both ATP and NAD^+^) are highly expressed on endothelial and inflammatory cells.^8^, ^9^ In turn, endothelial disruption and ongoing inflammation are characteristic of the development of many diseases.^10^, ^11^ Moreover, impairment of extracellular nucleotide catabolism, such as ATP and ADP, and adenosine nucleoside catabolism and altered purinergic signalling are one of the components of the development of calcific aortic valve disease (CAVD), which is one of the most common cardiovascular diseases.^12^

The possible involvement of NAD^+^ and its metabolism in the development of valve dysfunction is still unclear and requires further studies because the definitive mechanism of CAVD is not well determined. It is a slowly progressing disorder with a mechanical and inflammatory background. At an early stage, it is an atherosclerosis‐like process, which includes inflammation, accumulation of oxidized lipoproteins and active mediators of calcification, for instance osteopontin.^13^ In the cardiovascular system, different types of cells such as endothelial cells (ECs), vascular smooth muscle cells (VSMCs), immune cells or platelets could release nucleotides to the extracellular space where they act as signalling molecules by the stimulation of purinergic receptors.^14^ Extracellular metabolism of adenine nucleotides plays a crucial role in the regulation of pathological processes including inflammation, endothelial dysfunction or calcification, both in atherosclerosis and in CAVD.^15^ The deletion of CD73, responsible for the hydrolysis of AMP, NAD^+^ and NMN, leads to aortic valve dysfunction in mice. Mice have a similar effect as after a high‐fat diet. Thickening of the aortic valve leaflets, mineralization and disruption of valve function, which are hallmarks of aortic stenosis, occurs.^16^ Other deletion effects in mice include dyslipidaemia and intracellular lipid accumulation in muscle,^17^ impairment of tubuloglomerular feedback regulation of GFR,^18^ and altered thromboregulation and augmented vascular inflammatory responses.^19^ Moreover, mutations in the NT5E gene, which encodes CD73, lead to disturbances of extracellular nucleotide metabolism and progression in ectopic tissue calcification in humans.^20^, ^21^

Some aspects of NAD^+^ and its derivatives metabolism, their role in the extracellular space as a component of signalling processes via the NAD^+^ are still unclear and require further investigation. Considering that NAD^+^ is also a nucleotide present in the extracellular space like ATP, and the metabolic pathways of the two compounds are linked, we want to assess for the first time the extracellular degradation of NAD^+^ and NMN on the surface of vessels obtained from CD73−/− mice and pathologically altered aorta and aortic valves in humans.

## 2 MATERIALS AND METHODS

### 2.1 Reagents and antibodies

Unless otherwise specified, all reagents and chemicals were obtained from Sigma‐Aldrich. Nicotinamide riboside was obtained from Carbosynth. Antibodies for human CD38, CD73, ALP and eNPP‐1 were from Novus Biologicals. Fluorescent‐conjugated secondary antibodies were from Jackson ImmunoResearch Laboratories.

### 2.2 Human material

All experiments were performed based on the standards of the Declaration of Helsinki. The study was approved by the Local Bioethical Committee at the Medical University of Gdansk (NKBBN/19/2018). The informed consent forms have been obtained from all patients. Patients with CAVD were characterized by altered echocardiographic parameters compared with the control group. There were no significant differences between the control and study group with regard to age, body mass index (BMI) and biochemical data, or in terms of accompanying diseases and drug supplementation (Table 1). Aortic valves were collected from patients undergoing Bentall procedure (n = 10) and aortic valve replacement (AVR) surgery (n = 50) at the Clinic of Cardiac and Vascular Surgery, Medical University of Gdansk, Poland. Additionally, fragments of the ascending aorta were collected from three patients who underwent Bentall surgery. The material was immediately transported to the laboratory in physiological saline, then rinsed in Hank's balanced salt solution (HBSS) and used in experiments.

**TABLE 1:** Characteristics of patients whose aortic valves were used to analyse the metabolism of NAD^+^ and its derivatives. Results are shown as mean ± SEM or percentage

Clinical variables	Control (n = 10)	CAVD (n = 50)	*P*‐value
Age (y)	64 ± 2	69 ± 1	NS
Sex (male, %)	100	56	**0.0001**
BMI (kg/m^2^)	27.4 ± 1.7	28.6 ± 3.8	NS
Biochemical data			
Fasting blood glucose (mg/dL)	104.6 ± 5.9	108.3 ± 3.8	NS
Total cholesterol (mg/dL)	152.0 ± 6.0	193.8 ± 8.0	NS
Triacylglycerols (mg/dL)	107.8 ± 25.4	121.2 ± 8.8	NS
LDL‐cholesterol (mg/dL)	76.0 ± 3.5	120.0 ± 8.3	NS
HDL‐cholesterol (mg/dL)	54.8 ± 3.5	51.5 ± 2.9	NS
eGFR (mL/min/1.73 m^2^)	75.5 ± 6.5	63.6 ± 2.6	NS
Echocardiographic data			
Aortic valve area, AVA (cm^2^)	1.08 ± 0.05	0.79 ± 0.02	**0.0008**
Peak aortic valve velocity, V_max_ (m/s)	3.0 ± 0.4	4.2 ± 0.1	**0.0001**
Aortic mean gradient (mmHg)	32.6 ± 2,3	45.3 ± 2.1	NS
Comorbidity (%)			
Hypertension	60	56	NS
Diabetes mellitus	10	22	NS
Chronic kidney disease	10	4	NS
Coronary artery disease	20	32	NS
Atrial fibrillation	30	26	NS
Medication (%)			
ACEIs/ARBs/MRAs	80	72	NS
β‐Blockers	60	68	NS
Diuretics	60	46	NS
Digitalis	30	8	NS
Statins	60	66	NS
Anticoagulants	50	60	NS

Abbreviations: ACEIs, angiotensin converting enzyme inhibitors; ARBs, angiotensin receptor blockers; BMI, body mass index; MRAs, mineralocorticoid receptor antagonists. P‐value in bold = statistically significant

### 2.3 Animals

All experiments were conducted following a Guide for the Care and Use of Laboratory Animals published by the European Parliament, Directive 2010/63/EU, and were performed with the approval of the Local Ethical Committee for Animal Experimentation in Bydgoszcz (4/2018). Three‐month‐old male knockouts for CD73 on the C57BL/6J background (CD73−/−) were obtained from Heinrich‐Heine‐Universität in Düsseldorf, Germany, and were bred in the house.^19^ Three‐month‐old C57BL/6J wild types (WTs) were used as controls. Animals had direct access to standard chow diet and water. Mice were anaesthetized with a mixture of ketamine (100 mg/kg) and xylazine (10 mg/kg) given intraperitoneally. After opening the chest in each mouse, the aorta was removed. Fragments of the aorta were placed into cold physiological saline and purified from the surrounding tissues. Each fragment was cut along to expose the inner side and used for the measurements of ecto‐enzyme activities.

### 2.4 Determination of the extracellular NAD^+^ and NMN catabolism on the surface of human aortic valves and mouse vessels

The fibrosa surface of human aortic valves and fragments of mouse aorta was exposed using the procedure described previously.^15^ Valves and vessels were incubated with NAD^+^ and NMN in a final concentration of 50 µmol/L in 1 mL of HBSS, respectively. HBSS buffer at pH 7.35 was composed of 1.3 mmol/L CaCl_2_ × 2H_2_O, 0.4 mmol/L KH_2_PO_4_, 5.4 mmol/L KCl, 0.8 mmol/L MgSO_4_ × 7H_2_O, 0.1 mmol/L NaH_2_PO_4_ × 7H_2_O, 0.14 mol/L NaCl and 4.2 mmol/L NaHCO_3_ with 5.6 mmol/L glucose added before analysis. The medium was changed after each incubation, and fragments of the aorta and aortic valves were washed twice with buffer. Samples in the amount of 50 µL were collected after 0, 30, 60 and 120 minutes of incubation at 37°C. Before the analysis, samples were centrifuged (14 000 *g*/15 min/4°C) and metabolic changes were tracked by reversed‐phase high‐performance liquid chromatography (RP‐HPLC). Data were shown as nmol/min/cm^2^.

### 2.5 Determination of particular ecto‐enzymes engaged in the extracellular NAD^+^ and NMN catabolism

To determine the specific enzymes engaged in the formation of NAD^+^ and NMN human aortic valves (n = 9), fragments of ascending aorta (n = 9) and mouse vessels obtained from WT (n = 10) and CD73−/− mice (n = 8) were divided into smaller portions. The analysis was carried out as described previously.^15^ Briefly, aortic valves, purified fragments of human vessels and mouse aorta were preincubated (15 minutes) in 1 mL of HBSS with specific inhibitors of enzymes potentially involved in extracellular NAD^+^ and NMN degradation: competitive inhibitor of CD38, namely 150 µmol/L nicotinamide hypoxanthine dinucleotide (deamino‐NAD^+^), CD73 inhibitor—50 µmol/L AOPCP, 50 µmol/L PPADS as an eNPP1 inhibitor, and ALP inhibitor—500 µmol/L levamisole. NAD^+^ and NMN were added as substrates at a final concentration of 50 µmol/L, respectively. After 0, 30, 60 and 120 minutes, 50 µL of the sample was collected, centrifuged (14 000 *g*/15 min/4°C) and analysed by RP‐HPLC. In the case of human tissue, the degradation rates were normalized to the surface area. In mice, the reaction area was estimated using ImageJ software (National Institute of Health, USA) and final data were shown as nmol/min/cm^2^. Zukowska et al showed that the addition of AOPCP to the assay did not further reduce the concentration of reaction products.^16^ Therefore, in our experiment, we did not add this inhibitor by studying NAD^+^ and NMN hydrolysis in CD73−/− mice.

### 2.6 Analysis of the human aortic valve composition by histological staining and cellular sources of ecto‐enzymes by immunofluorescence

Aortic valves were stained using haematoxylin/eosin (HE) and Masson's trichrome (TR) staining, as well as the previously published Orcein Martius Scarlet Blue (OMSB) protocol, to distinguish collagen, elastic lamina and smooth muscle cells and visualize atherosclerotic plaques in valves obtained from CAVD patients.^22^ To distribute and localize CD38, CD73, eNPP1 and ALP protein, we used immunohistochemistry and antibody conjugated to a fluorophore (immunofluorescence) in human non‐stenotic (n = 4) and stenotic aortic valves (n = 3) as described previously.^23^ Briefly, fragments of tissue were completely embedded in the OCT compound, frozen in −80°C and then cut by a cryostat. Prior to analysis, the cut sections were thawed, placed in a blocking buffer, then applied primary and secondary antibodies according to the manufacturer's instructions. Calculation of the surface area of the fluorescence signal corresponding to a specific ecto‐enzyme and image analysis were performed using the ImageJ program. The cut‐off was set as the fluorescence intensity limit for a given signal.

### 2.7 Statistical analyses

The values were presented as a mean ± SEM. Normality was assessed using the Shapiro‐Wilk normality test. All parameters were tested by unpaired t test, Mann‐Whitey test or one‐way analysis of variance followed by the Tukey test with the use of GraphPad Prism 5 (GraphPad Software, San Diego, CA). Correlation was estimated by the Pearson r test. *P*‐value < 0.05 was considered significant.

## 3 RESULTS

### 3.1 Detection of active extracellular NAD^+^ and NMN degradation in WT and the CD73−/− mice

**FIGURE 2:**
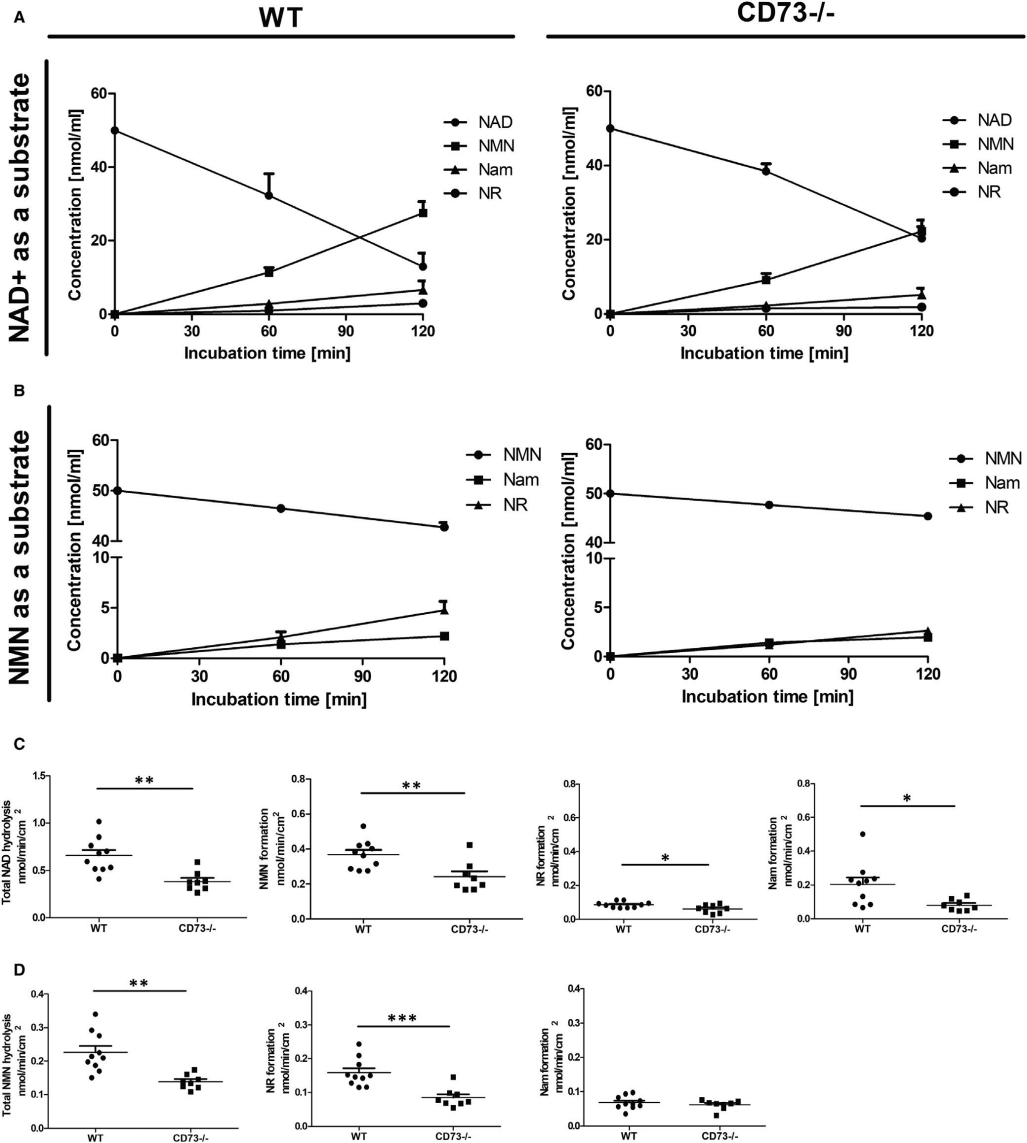
Degradation of NAD^+^ and NMN on the aortic surface and its inhibition in CD73−/− mice. The concentration of substrates and products after 120 min of incubation with 50 μmol/L NAD^+^ (A) and NMN (B) on the surface of the mouse aorta in HBSS. Results are shown as mean ± SEM; n = 8‐10. The rate of hydrolysis of NAD^+^ (C), hydrolysis of NMN (D) and product formation on the aortic surface of CD73−/− mice. Results are shown as mean ± SEM; n = 8‐10 *P < 0.05; **P < 0.01; ***P < 0.001 vs WT by unpaired Student's *t* test

The active NAD^+^ degradation and NMN degradation were still observed in CD73−/− mice, but it was reduced in comparison with WT mice (Figure 2A,B). NMN, as well as NR formation, was significantly lower in CD73−/− mice, both in the case of NAD^+^ degradation and in the case of NMN degradation. Nam production was significantly reduced only in the case of NAD^+^ degradation (Figure 2C). There was no difference between the production of Nam from NMN in both groups of mice (Figure 2D).

### 3.2 CD73 is not the only enzyme involved in the degradation of NMN to NR

**FIGURE 3:**
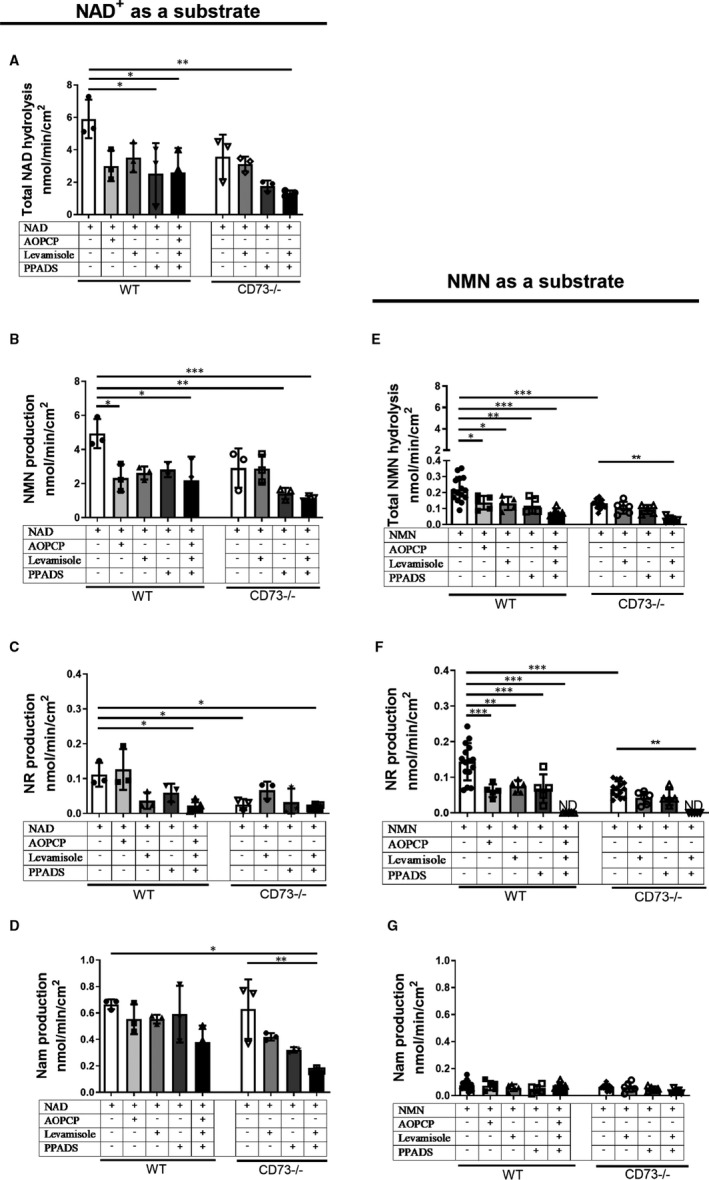
CD73, ALP and eNPP1 are the main enzymes involved in the catabolism of NAD^+^ and NMN to NR in mice. The total rate of NAD^+^ hydrolysis (A), the formation of NMN (B), the formation of NR (C), the formation of Nam (D), the total rate of hydrolysis of NMN (E), the formation of NR (F) and formation of Nam (G) on the surface of the murine WT and CD73−/− in the presence of specific ecto‐enzyme inhibitors. Results are presented as mean ± SEM; n = 8‐10; *P < 0.05, **P < 0.01, ***P < 0.001 vs without ecto‐enzyme inhibitors with one‐way ANOVA followed by Tukey's post hoc test

Inhibition of CD73, eNPP1 and ALP resulted in a significant reduction in total NAD^+^ degradation both in WT and in CD73−/− mice (Figure 3A). The increase in NMN (Figure 3E), NR (Figure 3C) and Nam (Figure 3G) production was reduced after inhibition of CD73, eNPP1 or all three enzymes tested simultaneously.

CD73 was the main enzyme involved in the degradation of NMN to NR. ALP and eNPP1 were responsible for the remaining hydrolytic activity (Figure 3B,F). In the case of NMN hydrolysis to Nam, the tested inhibitors show that selected enzymes did not participate in this process (Figure 3D).

### 3.3 Extracellular NAD^+^ and NMN but not Nam are actively catabolized on the surface of human aortic valves

**FIGURE 4:**
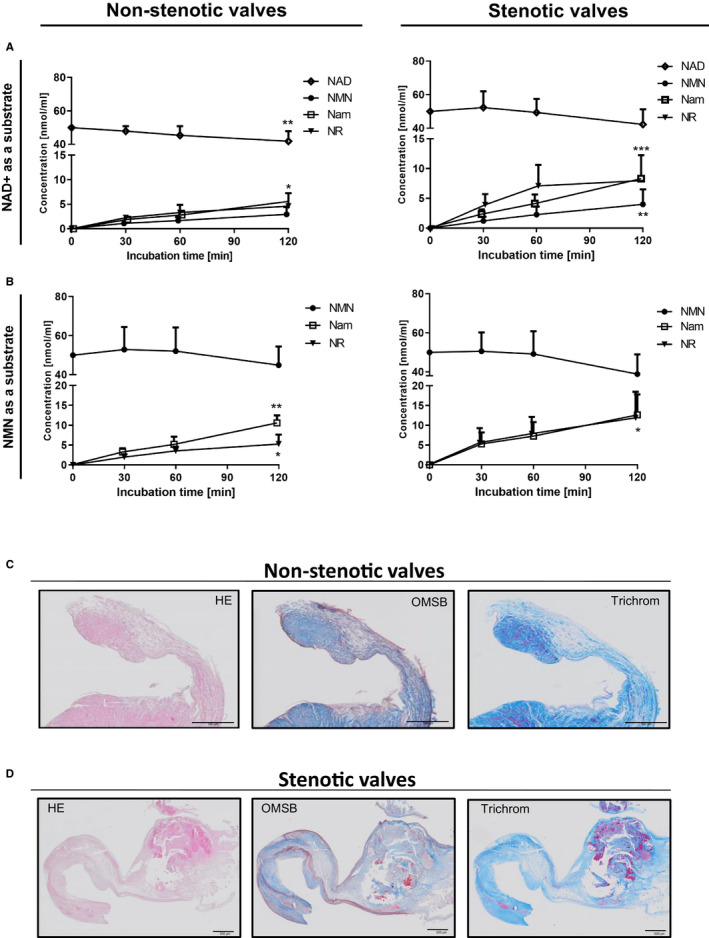
Active degradation of NAD^+^ and NMN is present on the surface of human non‐stenotic and stenotic aortic valves. Concentration of substrates and products after 120 min of 50 μmol/L NAD^+^ (A) and NMN (B) incubation of non‐stenotic (n = 10) and stenotic (n = 50) aortic valves in HBSS. Representative images of non‐stenotic (C) and stenotic (D) aortic valves (n = 3‐4) stained with haematoxylin and eosin (HE), orcein and martius scarlet blue (OMSB) and Masson's trichrome (TR); results are present as mean ± SEM

For the first time, the active degradation of extracellular NAD^+^ and NMN was observed on the surface of human aortic valves (Figure 4A,B). There was a lack of Nam and NR metabolism (data not shown). The main products of NAD^+^ degradation were NMN, Nam and NR. In turn of NMN degradation, Nam and NR were the primary products. The histological characteristics of non‐stenotic and stenotic valves are shown in Figure 4C,D, respectively. In recently described OMSB staining, the cell nuclei stained red, while the violet‐grey sections represent elastic fibres and elastic plaques, blue sections represent collagen fibres, and red nodules represent calcium deposits. In TR staining, cell nuclei were stained dark pink/red, dark blue sections show collagen fibres (dense connective tissue), light blue sections show extracellular matrix fibres (loose connective tissue), purple nodules represent calcium nodules, and red cells represent myofibroblast‐like cells. HE staining was used for general microscopy. Histological examination allowed the selection of valve fragments that were subjected to IF analysis.

### 3.4 CAVD reduces CD73 but stimulates the CD38 pathway

**FIGURE 5:**
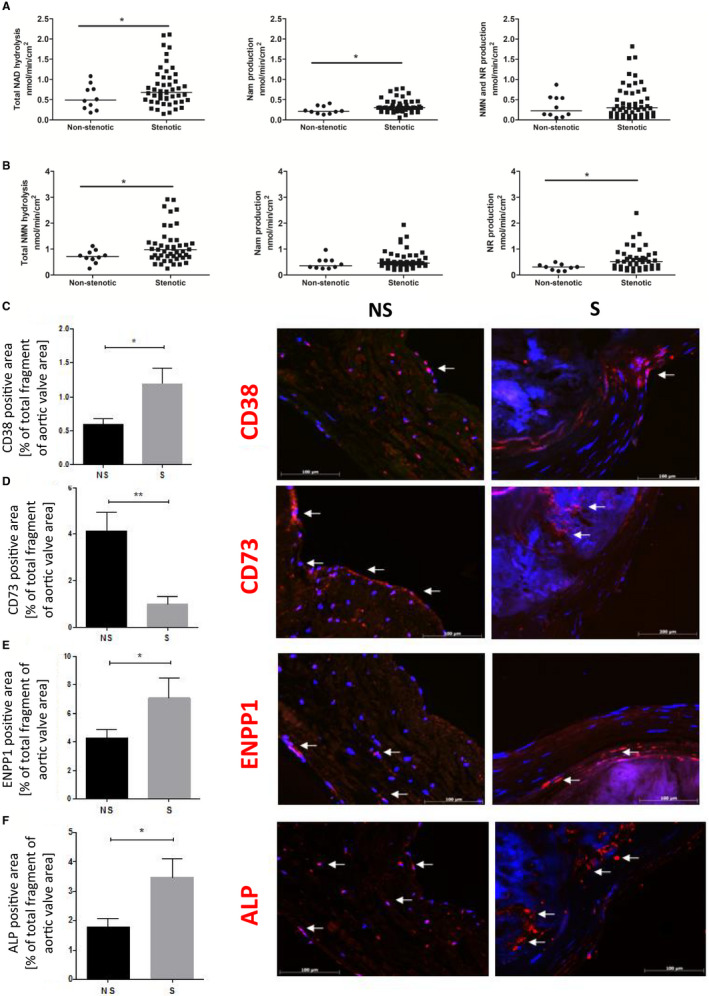
The signal for NAD^+^‐ and NMN‐degrading ecto‐enzymes (CD38, eNPP1, ALP) on the valve surface is increased in patients with CAVD, unlike CD73. The rate of hydrolysis of NAD^+^ (A), hydrolysis of NMN (B) and product formation on the surface of fibrosa of the aortic non‐stenotic (n = 10) and stenotic (n = 50) valves. The mean nucleotide conversion factor for each valve was estimated based on measurements for three leaflets independently at sites free of calcification. Results are presented as mean ± SEM; *P < 0.05 vs non‐stenotic valve with the Mann‐Whitney test. Representative images of matching immunofluorescence‐stained sections (red signal) for CD38 (C), CD73 (D), eNPP1 (E) and ALP (F). Quantitative analysis of the positive area for CD38, CD73, eNPP1 and ALP, which corresponds to a specific signal for each enzyme. Results are presented as mean ± SEM. NS, non‐stenotic; S, stenotic

CAVD patients were characterized by significantly increased NAD^+^ and NMN degradation on the surface of calcified valves compared with activity on non‐calcified valves (Figure 5A,B). There were no significant changes in NMN and NR production in NAD^+^ catabolism, while Nam production was higher in patients with CAVD. In the case of NMN metabolism, the formation of NR was almost twice as high in patients with CAVD as compared to the control group.

### 3.5 Decreased presentation of CD73 and increased presentation of CD38, eNPP1 and ALP in stenotic valves

The distribution of CD38, CD73, eNPP1 and ALP was analysed using immunofluorescence (IF). The CD73‐positive area was much smaller in stenotic valves in comparison with non‐stenotic valves (Figure 5D). Calculated data for the CD38, eNPP1 and ALP‐positive area and percentage of the total fragment of aortic valve area occupied by the studied enzymes showed a significant increase in stenotic valve responses compared with non‐stenotic valves (Figure 5C‐F).

IF analysis of stenotic valves revealed that CD38 was mainly located on the inner surface, directed to the calcification component, and a part of the CD38 signal was visible within the endothelium of aortic side (Figure 5C). The signal for CD73 was slightly visible in the inner side of the valve and was not observed on the endothelium (Figure 5D). eNPP1 and ALP were located within a calcified site (Figure 5E,F). The inner side is considered as spongiosis, the area of calcium deposits and some of the interstitial cells.

### 3.6 CD38 is a predominant enzyme responsible for nicotinamide formation from NAD^+^, whereas CD73 is largely involved in NMN degradation on the surface of human aortic valves

**FIGURE 6:**
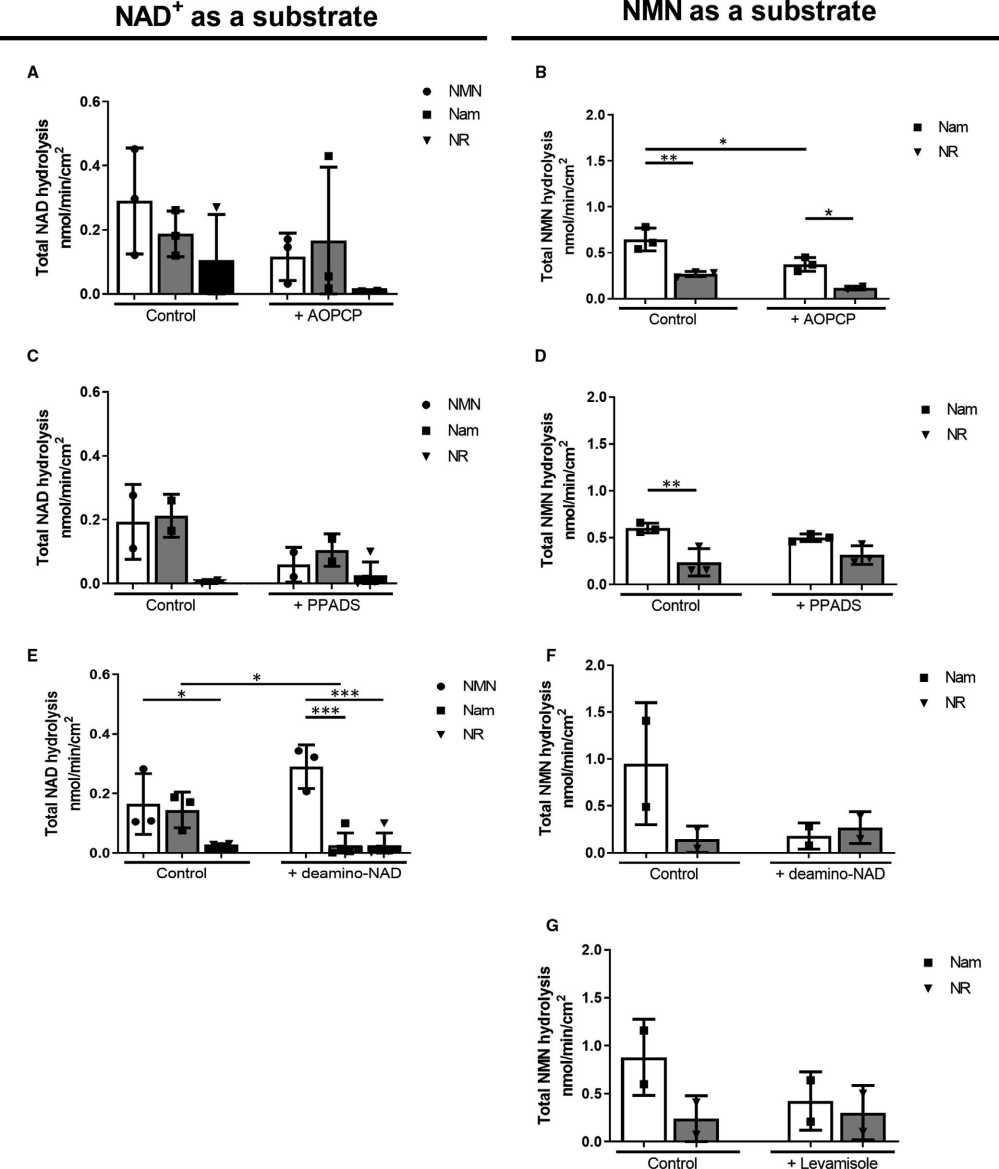
Inhibition of CD38 significantly reduces Nam production in the case of NAD^+^ catabolism, while CD73 inhibited by AOPCP can be mainly involved in NMN degradation on the surface of aortic valves. Total catabolism of NAD^+^ (A, C, E) and NMN (B, D, F, G) on the surface of human non‐calcified aortic valves in the presence of inhibitors of ecto‐enzymes: AOPCP (A, B), PPADS (C, D), deamino‐NAD^+^ (E, F) and levamisole (G). Results are presented as mean ± SEM; n = 9; *P < 0.05, **P < 0.01, ***P < 0.001 vs without ecto‐enzyme inhibitors by ANOVA followed by Tukey's post hoc test

Specific inhibitors were used to inhibit NAD^+^‐ and NMN‐degrading enzyme activity (the deamino‐NAD^+^ as a CD38 inhibitor, AOPCP as a CD73 inhibitor, PPADS as an inhibitor of eNPP1 and levamisole inhibiting ALP activity). This study has shown that CD38 (Figure 6E), and to a lesser extent eNPP1 (Figure 6C) and CD73 (Figure 6A), had the involvement in the degradation of NAD^+^. Moreover, inhibition of CD38 activity abolished Nam production and resulted in increased NMN formation (Figure 6E). In the case of NMN catabolism, the total NMN hydrolysis and formation of NAM were significantly decreased in the presence of AOPCP (Figure 6B). Also, there was a trend in diminished NMN hydrolysis by PPADS, deamino‐NAD^+^ and levamisole (Figure 6D,F,G).

**FIGURE 7:**
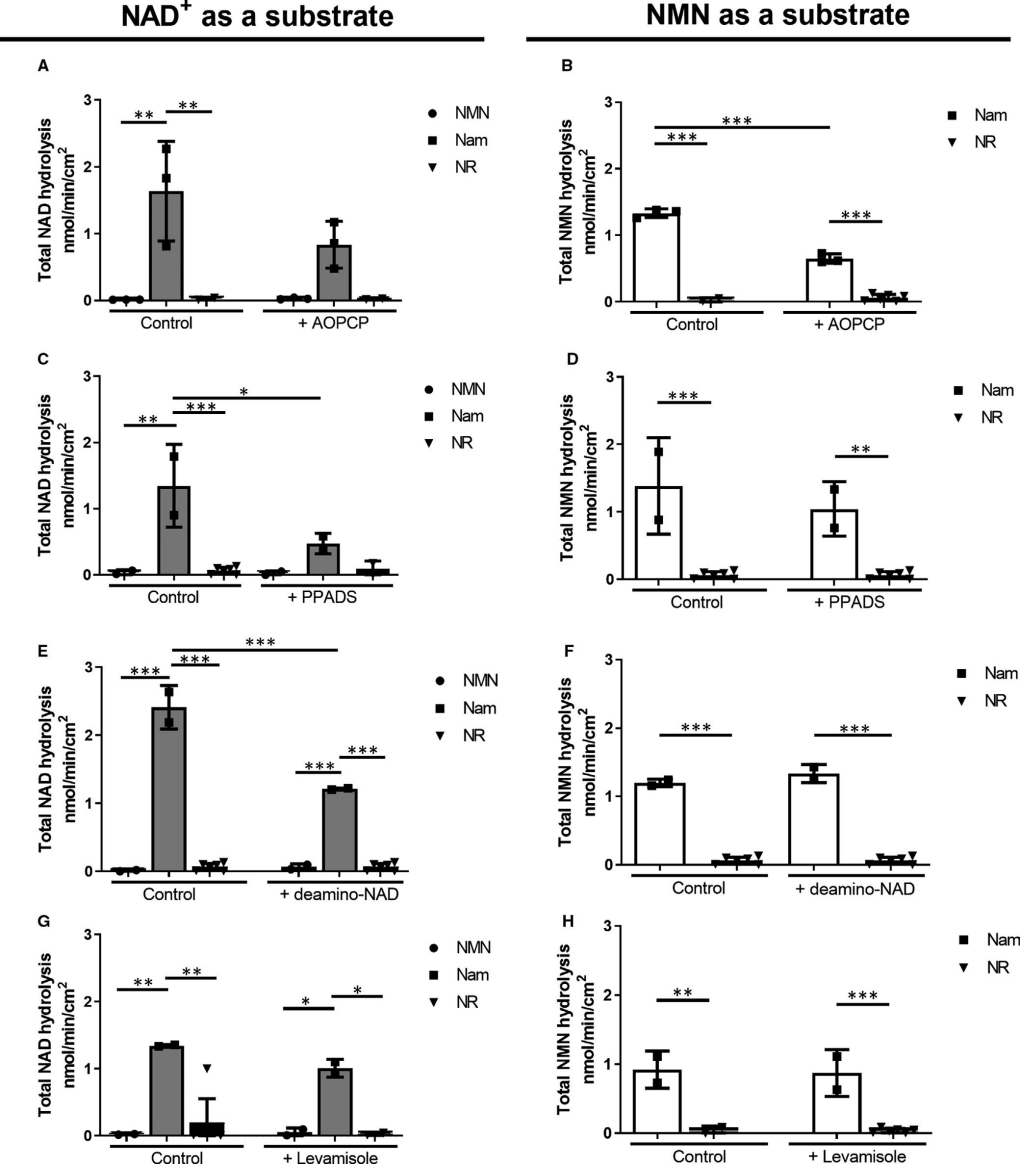
Nicotinamide is the major product of the degradation of both NAD^+^ and NMN on the surface of the human aorta. Total catabolism of NAD^+^ (A, C, E) and NMN (B, D, F, G) on the surface of the unaltered human aorta in the presence of ecto‐enzyme inhibitors: AOPCP (A, B), PPADS (C, D), deamino‐NAD^+^ (E, F) and levamisole (G, H). Results are presented as mean ± SEM; n = 9; *P < 0.05, **P < 0.01, ***P < 0.001 vs without ecto‐enzyme inhibitors by ANOVA followed by Tukey's post hoc test

Nicotinamide was the major product of NAD^+^ and NMN metabolism on the aorta surface (Figure 7). We observed significantly reduced NAD^+^ degradation after inhibition by PPADS and deamino‐NAD^+^, respectively (Figure 7C, Figure 7E), but not in the case of ALP inhibition (Figure 7G). AOPCP, as a CD73 inhibitor, significantly reduced the formation of Nam from NMN (Figure 7B), while the degradation of NAD^+^ by this enzyme did not change on the surface of the human aorta (Figure 7A). No significant changes in eNPP1, CD38 and ALP activity were observed after inhibition (Figure 7D,F,H).

## 4 DISCUSSION

This study is the first to demonstrate that extracellular degradation of NAD^+^ and NMN occurs and is very active on the surface of human aortic valves and the surface of human and mouse aorta. In addition, we observed altered metabolic patterns represented by decreased CD73 expression and increased CD38 expression in CAVD patients.

NAD^+^ enters in the extracellular space from lysed of damaged cells, or it can also be released by non‐cell destructive mechanisms, involving connexin 43 hemichannels.^24^, ^25^ Under normal conditions, released NAD^+^ is removed from the extracellular space by ecto‐enzymes. NAD^+^ and its derivatives hydrolysis may involve several enzymes, including CD73, CD38 and eNPP1, and non‐specific ALP.^26^, ^27^, ^28^ In our research, we proved the presence of these enzymes on the surface of the mouse aorta, as well as on human tissue samples, which are aortic valves and ascending aorta fragments in biochemical analysis. CD73−/− mice had reduced NAD^+^ and NMN degradation rate indicating a significant but not the only contribution of CD73 to the catabolism of NAD^+^ and its precursor. We have shown that eNPP1 and ALP are responsible for the remaining NAD^+^ and NMN catalytic activity. This is in line with previous studies published by our group about the contribution of CD73 to AMP hydrolysis.^9^ Allowedly, CD73 is primarily responsible for the formation of adenosine. The Km value of CD73 for AMP is in the low micromolar range, and the affinity of CD73 for AMP is considerably higher than for NAD^+^ or NMN.^5^ Nevertheless, in our study, inhibition of this enzyme by AOPCP or deletion of CD73 in mice resulted in an approximately twofold reduction in NAD^+^‐degrading activity, as well as an approximately threefold reduction in NR formation in NMN in mice. These studies indicated an important role of CD73 in extracellular NAD^+^ metabolism.

Moreover, our research has shown a significant decrease in the positive expression of the CD73 signal in the IF study on the stenotic valves in patients with CAVD. Reduced CD73 expression may be associated with the presence of inflammation and contribute to the development of aortic valve dysfunction.^16^ This is also consistent with publications where mutations in the NT5E gene, which encodes CD73, lead to altered extracellular nucleotide metabolism and progression in ectopic tissue calcification in the CAVD patient cohort.^20^, ^21^ In contrast to these results, it has been reported that stenotic aortic valves can exhibit overexpression of CD73.^29^

The presence of intensified NAD^+^ and NMN metabolism in patients with CAVD in our study can be confirmed by the observed significant increase in the expression response of IF analysis to other proteins involved in NAD^+^ hydrolysis, namely CD38, eNPP1 and ALP, as well as biochemical analysis. Additionally, our research indicated that after inhibition of CD38 activity on the aortic valve surface, there was a significant decrease in NAD^+^ catabolism. It is well known that CD38 contributes to the regulation of metabolism at the physiological level and is involved in the pathogenesis of multiple conditions, including ageing, heart disease and inflammation.^8^, ^30^, ^31^, ^32^ Increased CD38 activity in the cardiovascular system can be the result of many factors. In particular, the pro‐inflammatory state observed during the ageing process may provide CD38 expression by inflammatory cytokines, endotoxins, interferon and some nuclear receptors.^33^, ^34^ It has been shown that IFN‐γ may be responsible for the up‐regulation of CD38 and the increased adhesion of monocytes to ECs.^35^ In addition, angiotensin II (Ang II), which induces the production of reactive oxygen species (ROS), activates apoptotic signalling pathways and promotes inflammation, thrombosis and disruption of endothelial cells, may be responsible for activating CD38 in smooth muscle cells and cardiomyocytes.^36^, ^37^ Boslett et al showed that endothelial cells have a high expression of CD38, which is activated by hypoxia‐reoxygenation, causing CD38‐mediated NADP(H) depletion.^8^ Our research proved the significant contribution of CD38 to NAD^+^ metabolism on the surface of aortic valves and its increased distribution in patients with CAVD. According to our research, recently published work has shown that CD38 is responsible not only for NAD^+^ catabolism but also for the degradation of NAD^+^ precursors before they can be incorporated into the cell to rebuild the intracellular NAD^+^ pool.^27^, ^30^ Nevertheless, the exact cellular origin of CD38 should be investigated. We have previously published a work showing the exact origin of the CD73 activity in the cytometric analysis.^23^

On the other hand, CD38, which has two catalytic activities (NAD^+^‐glycohydrolase and ADP‐ribosyl cyclase) can play the role of cyclase in the formation of NAD^+^‐derived calcium messengers in physiology and pathology, which has been extensively studied.^38^, ^39^, ^40^, ^41^ Although the NAD^+^ function of glycohydrolase (ecto‐NADase) is predominant,^42^, ^43^ our further research is needed to investigate this aspect in the development of CAVD.

Importantly, CD38 not only regulates extracellular NAD^+^ homeostasis but also modulates the availability of extracellular NAD^+^ and its metabolites.^30^, ^44^ Thus, CD38 ecto‐NADase activity plays a role in the extracellular pathway of adenosine.^45^ In fact, it has been reported that extracellular adenosine can be produced by both NAD^+^ and ATP degradation by a cascade of events that include CD38, CD39 and CD73 activity. The recent study of our group demonstrated that disorders in vascular extracellular adenosine metabolism in mice correlate with susceptibility to atherosclerosis.^46^ Furthermore, patients with CAVD were characterized by specific adenine nucleotide ecto‐enzyme metabolic patterns.^23^ We observed reduced ATP and AMP hydrolysis and increased the deamination of adenosine. Mahmut et al who have a completely different point of view suggest that adenosine from AMP promotes mineralization valves in CAVD patients through the A2a adenosine receptor.^29^ The divergence with our conclusions can be a consequence of the use of only selected areas of pathologically altered valve. In our IF study, it can be seen that the signal for CD73 on the stenotic valve is only observed around the calcium deposits, and not on the entire surface of the endothelium as it is noticeable on non‐stenotic valves. Nevertheless, enzymes involved in the extracellular nucleotide cascade play a significant role in all stages of CAVD development, from controlling endothelial damage, through leucocyte infiltration, accumulation of foam cells and secretion of pro‐inflammatory mediators to osteoblastic differentiation. Our previous studies have shown that ATP and AMP hydrolyses negatively correlate with the concentration of calcium, magnesium and phosphate deposits in aortic stenotic valves, while the rate of adenosine degradation tended to negative correlation with calcium and phosphate deposits.^23^ Moreover, increased levels of calcium, magnesium and phosphate were observed in CD73‐deleted mice.^16^

Lower adenosine levels, as a result of reduced CD73 activity, may affect the decline in suppression of tissue–non‐specific ALP. This enzyme is related to the regulation of intracellular calcification.^47^ CD73 depletion in terms of both expression levels and enzyme activity is linked with ALP up‐regulation in cardiomyocyte hypertrophy.^47^ In our study, we observed increased signal for ALP in an IF study of stenotic valves and diminished hydrolysis of NMN after ALP inhibition on the surface of human valves. On the other hand, an extracellular inorganic pyrophosphate (ePPi), which is a substrate for ALP and known inhibitor of calcification, is generated by eNPP1, another important enzyme in NAD^+^ degradation.^48^ Villa‐Bellosta et al have been reported that smooth muscle NPP1 and ALP control vascular calcification through effects on synthesis and hydrolysis of ePPi.^49^ We demonstrated that the immunofluorescence signal for eNPP1 was abundant within calcification areas, which is consistent with the observed high level of eNPP1 in valvular interstitial cells during CAVD where it could modulate vascular calcification processes.^50^

In our studies, we have shown that the surface of the human ascending aorta differs in the metabolism of NAD^+^ and NMN. The major product of NAD^+^ and NMN conversion was nicotinamide. Inhibition of CD38 and eNPP1 activity resulted in a significant decrease in Nam formation in NAD^+^ degradation. Modest amounts of NMN produced may indicate a significant role of CD38 on the aortic surface, which is responsible mainly for the formation of nicotinamide. In contrast, the use of AOPCP to inhibit CD73 resulted in a significant reduction in the formation of Nam from NMN. This highlights a unique metabolic pattern on the surface of the aortic valve.

Nikiforov et al provide that bases (NA and Nam) and nucleosides (NR and NAR) are taken up by human cells to restore intracellular NAD^+^ pool, but not nucleotides (NAD^+^ and NMN).^51^ Additionally, Nam and NR could be converted to NAD^+^ by nicotinamide phosphoribosyltransferase (NAMPT) and nicotinamide mononucleotide adenylyltransferase (NMNAT) in the cells.^30^, ^51^ The lack of metabolism of Nam and NR on the surface of the aortic valves may be evidence of the lack of NAD^+^ resynthesis in the extracellular space (data not shown). However, the presence of eNAMPT, which catalyses the conversion of nicotinamide to NMN, requires further study.^52^ There is some evidence that eNAMPT derived from monocytes in myocardial adaptation to pressure overload can play a possible role in controlling intervention of myocardial NAD^+^ against heart failure.^53^

It is worth underlining that the extracellular NAD^+^, all enzymes involved in its metabolism and products of their action can regulate the local immunological microenvironment. There have been reports that extracellular NAD^+^, released from injured cells or during inflammation, can induce cell death in vitro and in vivo models due to suppression of T‐cell functions.^54^, ^55^ Thus, it can serve to limit the destructive action of autoreactive T cells, infiltrating the site of tissue injury.^56^ Apart from the ability to induce P_2_X_7_R‐dependent T‐cell death, Hong et al showed that NAD^+^ also increases P_2_X_7_R sensitivity to ATP gating in macrophages based on ART2.1‐dependent manner.^57^ In addition, extracellular NAD^+^ has been reported as an agonist at P_2_Y_11_ receptors in human granulocytes, acting as a pro‐inflammatory cytokine.^58^ Investigating the mechanisms that can be observed in the CAVD development process requires further research. There is some evidence to suggest that CD38 has been shown to regulate chronic inflammatory diseases such as autoimmune arthritis via nuclear factor kappa‐B pathway (NF‐κB), and this regulatory molecule may be a new target in the treatment of autoimmune inflammation.^59^ Moreover, the activation of the canonical NF‐κB pathway is responsible for the selective regulation of pro‐inflammatory and thrombotic reactions in human atherosclerosis.^60^ On the other hand, Doran et al observed that increased activation of CaMKII in lesional macrophages is associated with advanced and symptomatic atherosclerotic disease in both humans and mice and that deletion of myeloid CaMKIIγ in atheroprone mice suppresses the development of these advanced plaque characteristics.^61^ However, published work indicates that aortic stenosis and atherosclerosis can vary significantly, for example in the case of mechanisms that lead to increased oxidative stress.^62^ Further research will verify whether the above pathways may contribute to the development of CAVD disease and examine whether the regulation of enzymes involved in the extracellular metabolism of NAD^+^ can be an opportunity to find new therapeutic targets.

In summary, the extracellular metabolism of NAD^+^ has multiway importance. On the one hand, NAD^+^ itself and its metabolites can modulate the inflammatory process. NAD^+^ may be an alternative source in the formation of the anti‐inflammatory adenosine. On the other hand, Nam and NR, which are produced by ecto‐enzymes, can enter the cell and rebuild the intracellular pool of NAD^+^. In the case of a small amount of these precursors, disturbances may occur not only in cell energy metabolism and redox reactions but also in reactions involving intracellular NAD^+^‐metabolizing enzymes such as PARP or SIRT. Many studies have identified a relationship between reduced SIRT1 activity and the development of CAVD and atherosclerosis.^63^, ^64^ The consequence of the differences in our results between the aorta and valve may be related to the intracellular ATP expenditure. The synthesis of the intra‐NAD^+^ pool from Nam is much less efficient due to more steps. In turn, resynthesis with NR is more preferable. Further research should elucidate the exact significance of changes in the valves and in the vessels during disease progression.

In conclusion, this study showed for the first time catabolism of the extracellular NAD^+^. Individual enzymes involved in NAD^+^ catabolism and their location within valves were identified as well. CAVD patients were characterized by a changed metabolic pattern on the surface of the calcified valve, which may indicate the unique role of NAD^+^‐depending ecto‐enzymes in CAVD. A precise description of the role of changes will help to better understand the processes leading to the development of CAVD and could be a promising target for therapy of vascular pathologies.

## CONFLICT OF INTEREST

The authors declare that they have no conflict of interest.

## AUTHOR CONTRIBUTIONS

**Patrycja Jablonska:** Data curation (lead); Formal analysis (lead); Investigation (lead); Methodology (lead); Project administration (lead); Visualization (lead); Writing‐original draft (lead); Writing‐review & editing (lead). **Barbara Kutryb‐Zajac:** Formal analysis (supporting); Investigation (supporting); Methodology (supporting); Software (supporting); Visualization (supporting); Writing‐original draft (supporting). **Paulina Mierzejewska:** Formal analysis (supporting); Investigation (supporting); Methodology (supporting); Resources (supporting). **Agnieszka Jasztal:** Methodology (supporting); Software (supporting); Validation (supporting). **Barbara Bocian:** Data curation (supporting); Resources (supporting). **Romuald Lango:** Data curation (supporting); Resources (supporting). **Jan Rogowski:** Data curation (supporting); Resources (supporting); Supervision (supporting). **Stefan Chlopicki:** Supervision (supporting). **Ryszard T Smolenski:** Conceptualization (supporting); Project administration (supporting); Writing‐original draft (supporting); Writing‐review & editing (supporting). **Ewa Maria Slominska:** Conceptualization (lead); Formal analysis (supporting); Funding acquisition (supporting); Project administration (lead); Supervision (lead); Writing‐original draft (supporting); Writing‐review & editing (supporting).

## Data Availability

Data are available on request due to privacy/ethical restrictions.
